# Powder Metallurgy Processing to Enhance Superelasticity and Shape Memory in Polycrystalline Cu–Al–Ni Alloys: Reference Material for Additive Manufacturing

**DOI:** 10.3390/ma17246165

**Published:** 2024-12-17

**Authors:** Mikel Pérez-Cerrato, Jose F. Gómez-Cortés, Ernesto Urionabarrenetxea, Isabel Ruiz-Larrea, Fernando Carreño, Ízaro Ayesta, María L. Nó, Nerea Burgos, Jose M. San Juan

**Affiliations:** 1 Department of Physics, Faculty of Science & Technology, University of the Basque Country, UPV/EHU, Apdo. 644, 48080 Bilbao, Spainmaria.no@ehu.es (M.L.N.); 2 CEIT-Basque Research & Technology Alliance, BRTA, Manuel Lardizabal 15, 20018 Donostia/San Sebastián, Spain; eugomez@ceit.es (E.U.); nburgos@ceit.es (N.B.); 3 Tecnun, Universidad de Navarra, Manuel Lardizabal 13, 20018 Donostia/San Sebastián, Spain; 4 Department of Physical Metallurgy, Centro Nacional de Investigaciones Metalúrgicas (CENIM–CSIC), Avda. Gregorio del Amo 8, 28040 Madrid, Spain; carreno@cenim.csic.es; 5 Aeronautics Advanced Manufacturing Centre, CFAA, University of the Basque Country, UPV/EHU, 48170 Zamudio, Spain

**Keywords:** shape memory alloys, Cu–Al–Ni, powder metallurgy, gas atomization, hot isostatic pressing, superelasticity, martensitic transformation

## Abstract

Shape memory alloys (SMAs) are functional materials with a wide range of applications, from the aerospace sector to the biomedical field. Nowadays, there is a worldwide interest in developing SMAs through powder metallurgy like additive manufacturing (AM), which allows innovative building processes. However, producing SMAs using AM techniques is particularly challenging because of the microstructure required to obtain optimal functional properties. This aspect is critical in the case of Cu–Al–based SMAs, due to their high elastic anisotropy, making them brittle in polycrystalline form. In this work, we approached the processing of a Cu–Al–Ni SMA following a specific powder metallurgy route: gas atomization of a pre-alloyed melt; compaction of the atomized powders through hot isostatic pressing; and a final hot rolling plus thermal treatments. Then, the microstructure of the material was characterized by electron microscopy showing a specific [001] texture in the rolling direction that improved the functional behavior. The successive processing steps produce an increase of about 40 °C in the martensitic transformation temperatures, which can be well controlled and reproduced through the developed methodology. The thermomechanical functional properties of superelasticity and shape memory were evaluated on the final SMA. Outstanding, fully recoverable superelastic behavior of 4.5% in tension, as well as a ±5% full shape memory recovery in bending, were reported for many cycles. These experiments demonstrate the enhanced mechanical and functional properties obtained in polycrystalline Cu–Al–Ni SMAs by powder metallurgy. The present results pave the road for producing this kind of SMA with the new AM technologies, which always produce polycrystalline components and can improve their processes taking the powder metallurgy SMA, here produced, as reference material.

## 1. Introduction

Additive manufacturing (AM) is the last generation of powder metallurgy (PM) technologies and emerged as a new paradigm in materials processing [1,2,3]. At present, the production of functional materials, such as shape memory alloys (SMA), is a challenge for AM, due to the well-controlled pores-free microstructure required to obtain good and reproducible functional properties. Indeed, the specific SMA thermomechanical properties of shape memory and superelasticity are associated with a diffusionless phase transition between a high-temperature (and high-symmetry) phase called austenite and a low-temperature (and low-symmetry) phase called martensite. This martensitic transformation (MT) is thermoelastic in SMAs and takes place through a reversible shearing of the atomic lattices, which is responsible for a noticeable change in shape that can reach up to 10% recoverable strain in single crystals [4,5]. The direct MT during cooling allows an easy change in shape, which is recovered by heating during the shape memory effect. Alternatively, during the superelastic effect (SE), at constant temperature, the MT is stress-induced by the application of an external stress promoting the lattice shearing and being responsible for a large superelastic strain, which is fully recovered when withdrawing the stress. These two properties allowed the development of a great number of technological applications in many industrial sectors, like robotic, aeronautic, civil engineering, and biomedical, among others [6,7]. However, the atomic shearing associated with the MT generates local strains and the development of a distributed field of experimentally measurable [8,9] internal stresses, which eventually could contribute to fissure propagation and premature fracture around microstructural defects and pores. In recent years, AM has faced this problem in SMAs, and many works have been devoted to optimizing the microstructure of Ni-Ti [10,11,12], Fe-based [13,14,15], and Ni-Mn-based [16,17,18] SMAs as well as Cu–Al–based SMA, which is the subject of our particular interest; a general review on SMAs produced by AM was recently published [19]. The interest in Cu–Al–based SMAs is motivated by different aspects: their capability of working at higher temperatures than the binary NiTi SMA [20,21]; their good behavior at the micro/nano scales in shape memory [22] and superelasticity [23,24], which makes this family of alloys excellent candidates for developing architected SMAs at different dimensional scales [25]; and also their mechanical damping performance [26]. Concerning the AM processing of Cu–Al–based SMA, some pioneering works were approached between 2015 and 2021 [27,28,29,30,31,32], which were focused on the processing parameters and general aspects of AM, including remelting through a double laser pass [30]. Then, a remarkable increase in interest was observed since 2022 in AM of Cu–Al–based SMAs looking for the optimization of the microstructure and mechanical properties [33,34,35,36,37,38,39,40,41]. Nevertheless, in order to establish the relationship between the AM processing parameters and the final functional performance, it is important to have a reference material for comparison. Obviously, this reference should not be the strongly textured polycrystalline wires of NiTi [42], nor the single crystal wires of Cu-based [21] SMAs, for instance, because they exhibit microstructures far different from the ones expected with AM processing. Thus, the reference material for AM performance comparisons should come from previous polycrystalline SMAs produced by PM processing routes.

**Figure 1:**
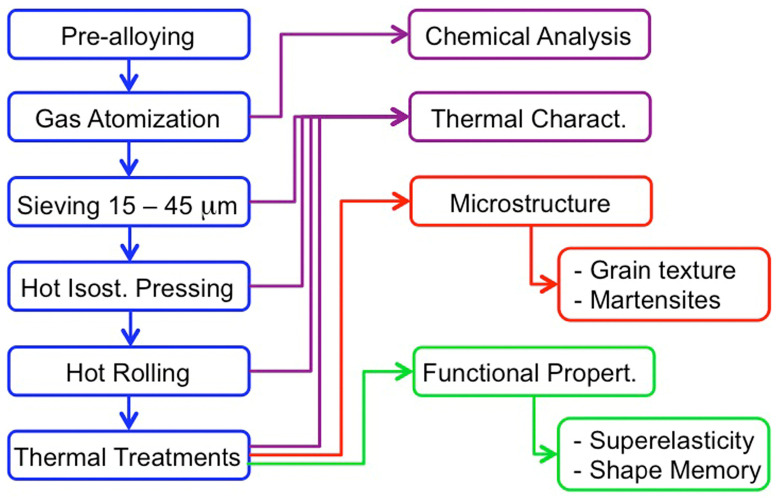
Flow chart of the complete processing procedure, in blue cases at the left, together with the characterizations performed along the study; microstructure in red and functional in green.

In this scenario, the goal of the present work is focused on a twofold objective. Initially, the state of the art of the powder metallurgy processing route applied to SMA is overviewed. A historical approach is presented, including the NiTi and Cu–Al–based SMA families, looking for the similarities and the differences. Then, from the lessons learned, a complete powder metallurgy processing route is proposed and experimentally developed. The thermal transformation and the microstructure are characterized, and the functional properties of the processed SMA are studied. The methodology is presented in the next section and in the schema of Figure 1, and it may be advanced that outstanding superelastic and shape memory behaviors were obtained. The final aim of this work is to show that the SMA processed by this PM route could constitute a reference material to evaluate the progress of the AM processing routes.

## 2. The State of the Art in Powder Metallurgy of Shape Memory Alloys

A general characteristic of SMAs is the strong dependence of the transformation temperatures (Ms-martensite start and Mf-martensite finish during cooling as well as As-austenite start and Af-austenite finish during heating) on the concentration of the solid solution elements of the alloy. Indeed, 1 wt% of Ni in NiTi SMA changes the transformation temperatures between 80 and 120 K [43], and 1 wt% of Al in Cu–Al–Ni SMA changes such temperatures by more than 170 K [44]. Furthermore, oxidation must be well controlled because oxygen will react with Ti (in NiTi) and with Al (in Cu–Al–based), depleting its concentration in solid solution and producing strong deviations in the transformation temperatures [4]. As a consequence, water atomization was soon discarded and the production of the powders was approached by gas atomization; see classic textbooks for a description of this technology [45,46,47]. Nevertheless, the motivations for using the powder metallurgy route instead of the traditional ingot metallurgy route are not the same for each family of SMA. However, these motivations are not described enough in a recent review on general processing methods in SMAs [48], and in this section, the motivation and the development of the powder metallurgy production route in the main SMA will be briefly overviewed.

### 2.1. Powder Metallurgy of NiTi Alloys

In the case of the Ni-Ti-based SMA families, the powder metallurgy route offered the attractive possibility of producing near-net-shape components to avoid the difficult machining process required in the ingot metallurgy route to obtain bulk components. The driving motivation was the interest in using Ni-Ti SMAs for biomedical applications [49,50]. Historically, due to the limited availability of gas-atomized powders of different Ni-Ti qualities, many works tried to produce bulk materials starting from mixed elemental powders and using several processing technologies, such as self-propagating high-temperature synthesis (SHS) [51,52], spark plasma sintering (SPS) [52,53,54], high-temperature sintering (HTS) [55,56,57], and hot isostatic pressing (HIP) [58,59,60], and in many cases, these techniques were applied to previously mechanically alloyed (MA) elemental powders [61,62,63,64,65,66,67,68]. However, the use of blended elemental powders makes it difficult to obtain a homogeneous microstructure and composition, and consequently, a good and reliable MT, as was already mentioned in early works [61]. Recent works on AM by electron-beam melting (EBM) [69] or by laser powder bed fusion (LPBF) [70] show that the use of elemental blended powders is not a good methodology to produce a functional Ni-Ti SMA. Alternatively, pre-alloyed NiTi powders were used through similar processing techniques like HIP [71,72,73], SPS [74], or even by metal injection molding (MIM) [60,75,76]. Nevertheless, a significant porosity was observed in most cases [77]. The transformation temperatures could be well controlled using these technologies, but the thermomechanical properties of shape memory and superelasticity are not usually reported because they may not exhibit good enough performance in comparison with that produced by the classic ingot metallurgy route [4,5] largely reported in bulk NiTi. Thus, in order to evaluate the progress, the fabrication of NiTi SMA components through AM [10,11,12] must still take as reference the shape memory and superelastic behavior previously obtained through the ingot metallurgy route [42,43].

### 2.2. Powder Metallurgy of Cu–Al–Based Alloys

In the case of Cu–Al–based SMAs, apart from the interest in producing near-net-shape components, there is another strong motivation for using the powder metallurgy route: to obtain a homogeneous microstructure with a fine grain size or, eventually, slightly textured to minimize the internal stresses between adjacent grains. Indeed, most of Cu–Al–based SMAs are Heusler alloys with a D0_3_ or L2_1_ atomic order exhibiting a high elastic anisotropy, which was observed by tensile tests on differently oriented single crystals of Cu-A-Ni [78] and determined through the measure of the elastic constants [79,80,81], showing an anisotropy factor A = 2·C_44_/(C_11_ − C_12_) ≈ 13; the elastic modulus, for instance, varies one order of magnitude between the [111] and [001] lattice directions. As a consequence, polycrystals of Cu–Al–based SMAs are very brittle, and during the required quenching from the high-temperature solid solution domain of the austenite (usually around 900 °C), the stress concentration at grain boundaries and triple points reaches very high values, producing intergranular fractures [82]. To solve this problem, several solutions were historically proposed to obtain SMAs with good performance. The first obvious solution was to use single crystals exhibiting exceptionally good shape memory and superelastic behavior, which have been studied for years by many research groups [78,83,84,85,86,87,88]. The second solution is looking in the opposite direction, and consists in decreasing the grain size in order to minimize the stress concentration at the grain boundaries. Different grain refiners are being used in Cu–Al–based SMAs, such as B and metallic elements like Ti, Zr, V [89,90,91,92,93], and recently, more exotic elements like Ce [94] and Gd [95]. However, the required solid solution high-temperature thermal treatment entails that the final refining effect is not as good as expected. Another solution to obtain fine-grained polycrystals in the Cu-based SMA was offered by powder metallurgy, and several technical solutions were approached. Many works used elemental powders that were blended by mechanical alloying, MA, followed by different compaction strategies: cold or hot preliminary compaction and a further HTS [96,97,98,99,100,101,102,103], hot sintering plus hot extrusion or rolling [104,105,106,107,108,109,110,111], as well as SPS [112]. Nevertheless, like in the case of Ti-Ni SMAs, the use of elemental powders through MA exhibits a rather high porosity [101] that, in the best of cases, is still about 1% [112]. In general, the use of elemental powders makes the control of the transformation temperatures difficult, and the final mechanical or functional properties are rather poor; in fact, none of the referred works [96,97,98,99,100,101,102,103,104,105,106,107,108,109,110,111,112] reported any superelastic behavior in the produced samples.

In parallel, other works used pre-alloyed powders that, in some cases, were cold compacted, sintered and hot-worked [113,114], or just hot-sintered at high pressure [115], but these preliminary works did not obtain good functional properties. Then, the use of pre-alloyed powders obtained by gas atomization followed by HIP compaction and hot rolling [116] succeeded in obtaining a 4% recoverable shape memory and a 1% fully recovered superelastic effect, which was extended later up to a 4% recoverable superelastic effect [117] with the same powder metallurgy methodology. Further developments by mechanical alloying of pre-alloyed powders of different compositions allowed a precise control of the transformation temperatures [118] and the texture [119]. However, MA introduces many oxide particles [119], and the final functional performances decreased with respect to the HIPed and rolled samples, obtaining a fully recoverable superelastic effect of 2% [120]. Recent works using pre-alloyed powders compacted by hot sintering [121] show that a higher porosity, about 10%, remains after sintering.

### 2.3. Conclusions from the Review on Powder Metallurgy of SMAs

From the above review of the state of the art in powder metallurgy of shape memory alloys, some lessons can be learned:

- Powders should be produced by gas atomization.
- The use of pre-alloyed powders is practically compulsory to obtain the required control and homogeneity of the martensitic transformation temperatures.
- The best compaction method seems to be hot isostatic pressing (HIP).
- Hot rolling breaks the oxide film surrounding the powder particles, improving the compaction and the texture of the material.
- The use of grain refiners in the pre-alloyed powders is recommended in order to avoid a large recrystallization during hot rolling and during the required standard solid solution thermal treatment.

Then, from the lessons learned along the last decades, the PM processing route will be used to develop a polycrystalline Cu–Al–Ni SMA, exhibiting high thermomechanical performance in both shape memory and superelastic effects, which could be taken as reference material for further works in PM and, in particular, in AM of Cu-based SMAs.

## 3. Materials and Methods

### 3.1. Experimental Methodology

The procedure developed to produce and characterize the SMA by powder metallurgy is described in Figure 1, which schematically presents the flow of the whole experimental methodology. Initially, the SMA was pre-alloyed and atomized by gas to obtain spherical powders, which were chemically analyzed. The powders were sieved to use the same size fraction (between 15 and 45 μm) usually employed in AM by laser powder bed fusion [1,2,10,11], so that the PM material could be taken as reference for the counterparts produced by AM. Then, the powders were compacted by HIP and further hot-rolled before undergoing the standard thermal treatments for functionalization. During this process, the thermal martensitic transformation was measured to follow the influence of the processing steps on the transformation temperatures. Although the microstructure was also tested along the different steps, special attention was devoted to the microstructure of the final SMA in order to associate it with the thermomechanical functional properties evaluated through superelastic and shape memory experiments.

### 3.2. Selected Alloy and Powder Preparation

The Cu_82.2_Al_13.3_Ni_4.5_ (wt.%) SMA was selected for the production of the samples, which has a nominal transformation temperature above room temperature [44]. Moreover, the alloy had an extra addition (above the target 100 wt.% composition) of 0.2 wt.% of B and 0.1 wt.% of Zr, which would act as grain refiners to improve the mechanical properties of the final samples. The alloy was produced using high-purity elements, adding the Ni, B, and Zr as master Cu–Ni, Cu–B, and Cu–Zr alloys, respectively, in order to reduce the melting point and, consequently, the losses of Al by evaporation and oxidation during the melting process. The control of the chemical composition of the alloy had to be extremely precise, because the transformation temperatures of this family of SMAs have a great dependence on Al content, producing a decrease of approximately 17 °C per 0.1 wt% of Al [21,44].

The alloyed powder was produced using gas atomization in a research atomization unit PSI model HERMIGA 75/3VI (Phoenix Scientific Industries Ltd., Hailsham, UK) of the CEIT. A description of the equipment is given in a previous work [122]. The whole melting process was performed under a high-purity Ar atmosphere and using Ar as the atomization gas at a pressure between 55 and 60 bars. Once the powders were obtained, the material was sieved to separate particles with grain sizes between 15 and 45 μm so that the powders had sizes similar to the ones used in AM by the laser powder bed fusion (LPBF) technique.

### 3.3. Chemical and Thermal Powders Characterization

The chemical composition of the atomized powders was measured by inductive coupled plasma optical emission spectroscopy (ICP–OES) in a Varian 725–ES ICP–OES equipment (Agilent Technologies, Santa Clara, CA, USA). Oxygen content was measured with a LECO TC–400, while carbon and sulfur impurities were measured using a LECO CS–200 (LECO Corporation, St. Joseph, MI, USA). The results are presented in Table 1, showing a low content of both O and C and with N and S appearing as residual impurities. Table 1 also shows the exceptionally good control of the composition during the atomization process.

**Figure 2:**
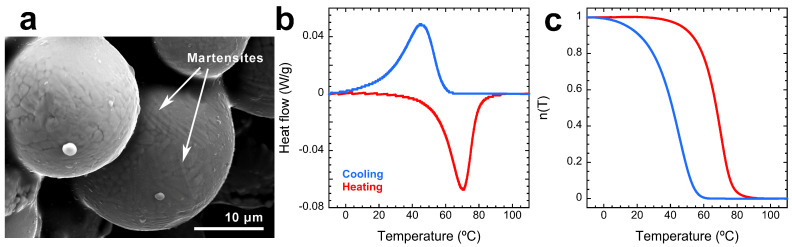
(**a**) SEM-SE image of a powder grain. The martensite variants can be appreciated on its surface in some of the grains. (**b**) DSC results for the powder material. (**c**) Phase-transformed fractions n(T) obtained by integration of the DSC results in (**b**), which are used for the determination of the MT temperatures.

The fast quench during atomization stabilizes the austenite phase at low temperatures, allowing the powders to undergo the MT, because for this composition the alloy is in a martensitic state at room temperature. The thermal transformation was measured by differential scanning calorimetry (DSC) using a Discovery DSC TA 2500 (TA Instruments, New Castle, DE, USA), with a temperature rate of 10 °C/min between a range of –20 and 120 °C, under a N_2_ atmosphere. Figure 2a shows a scanning electron microscopy (SEM) image of the atomized powders used for the fabrication of the samples. In this image, the presence of the martensites can be observed on the surface of the particles. Then, Figure 2b shows the DSC curve of the MT with the direct transformation on cooling (shown in blue) and the reverse transformation on heating (shown in red).

From the integration of these curves, the transformed martensite fraction n(T) can be derived, as shown in Figure 2c, which depicts the amount of material of each phase (martensite and austenite) that is present at different temperature values. By looking at the n(T) function, the transformation temperatures of the material can be obtained. In this regard, we chose the transformation temperatures as follows: n(T) = 0.02 (2% of martensite in the sample) marks the martensite start (Ms) and austenite finish (Af) temperatures, and n(T) = 0.98 (98% of martensite in the sample) marks the martensite finish (Mf) and austenite start (As) temperatures. By applying these criteria, the transformation temperatures for the Cu–Al–Ni powders are Ms = 57.2 °C, Mf = 6.6 °C, As = 39.0 °C, and Af = 82.6 °C. Additionally, the thermal hysteresis of the MT, ΔT, can be calculated as the difference between the values of n(T) = 0.5, which gives a value for ΔT = 26.4 °C.

### 3.4. Fabrication of the Powder Metallurgy Samples

**Figure 3:**
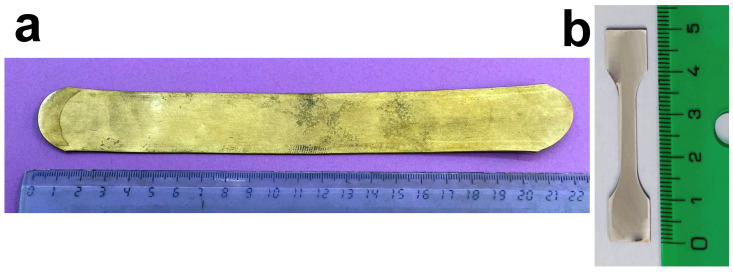
(**a**) Sample produced using a conventional method of powder metallurgy, HIP followed by hot rolling down to 1.1 mm thickness. (**b**) Dog-bone-shaped sample cut by EDM from the plate in (**a**), for the realizations of tensile mechanical tests.

The process of obtaining samples from atomized powders began by applying hot isostatic pressing (HIP) to the alloyed powders. The material was set at a temperature of 850 °C and underwent a pressure of 140 MPa for a fixed duration of 2 h. The material after HIP was processed by hot rolling (HR) at the facilities of the CENIM-CSIC. The HIP ingots were cut in plates with an initial thickness of 7.4 mm, which was reduced to 1.1 mm after 30 rolling passes, undergoing a thickness reduction of 6–8% on each rolling step. From these sheets, dog-bone samples for the mechanical tensile tests were cut using wire electrical discharge machining (WEDM) at the facilities of the CFAA in an ONA AV35 machine (ONA EDM S.A., Durango, Spain) under low-energy conditions. After wire cutting, the samples were hand-polished to remove the recast layer generated during the EDM process. An image of the HR samples is presented in Figure 3a, and an example of the final tensile sample in Figure 3b.

### 3.5. Thermal Treatments

The applied processing method destroys the austenitic phase, as it is not one of the stable phases of the Cu–Al–Ni phase diagram [123], and during the high-temperature processing, the precipitation of the stable phases takes place starting at the grain boundaries [124]. The high-temperature phase, called β_3_ austenite according to the standard nomenclature [125], is metastable at low temperatures and must be frozen to allow the further martensitic transformation. In order to restore the β_3_ austenite, a solid solution thermal treatment is required. To perform this treatment, the alloy is heated up to 900 °C inside an Ar atmosphere to prevent oxidation. At these temperatures, the austenite becomes the stable phase, and the rest of the phases dissolve into the austenite matrix. After 30 min to let the material reach equilibrium, the alloy is quenched in iced water, blocking the diffusion processes. Later, the alloy must be aged for 24 h at 180 °C in order to stabilize the atomic order and, therefore, the transformation temperatures [126]. All samples were subjected to this standard thermal treatment. The transformation temperatures and hysteresis were measured via DSC using the same method and equipment described for the atomized powders.

### 3.6. Microstructural Characterization Techniques

The microstructure of the samples was characterized by optical microscopy with Nomarski interferential contrast in a Leica DMRXA 2 (Leica Microsystems GmbH, Wetzlar, Germany) and in a SEM, FEG JEOL JSM–7000F (JEOL Ltd., Tokyo, Japan). Samples were ground and polished down to a particle size of 0.25 μm. Electron backscatter diffraction (EBSD) was used to identify the martensites using an Oxford-Aztec system (Oxford Instruments plc., Abingdon, UK). As the studied SMA has transformation temperatures above room temperature, see Figure 2, in order to characterize the texture of the austenite grains, it was necessary to heat the sample above the Af to revert the MT. Then, an EBSD in situ SEM experiment was performed by heating up to 145 °C, using the heating–cooling stage Gatan C1003 (Gatan Inc, from AMETEK Inc. group, Berwyn, PA, USA), allowing the characterization and texture analysis of the austenite grains. In all cases, the EBSD study was performed at 20 KV, 2 nA, 70° tilt, and 13.6 mm of working distance.

### 3.7. Thermomechanical Tests

The mechanical tests to study the superelastic behavior were performed in an Instron 4467 inside the Instron 3119–006 thermal chamber (Instron, Norwood, MA, USA). The strain was measured with an extensometer Instron of 10 mm gauge length. This setup allowed for the measurement of the SE at different temperatures to obtain a wide range of data in order to determine the Clausius–Clapeyron coefficient. A sample of dimensions 82 × 5 × 1 mm^3^ was cut from the same rolled plate shown in Figure 3a to evaluate the shape memory behavior. After the standard thermal treatment, the sample was heated at 135 °C to transform into austenite, and a three-point bending force was applied at this temperature. Then, while maintaining the bending force, the material was cooled down to martensite. This way, the material was deformed to a specific radius with respect to the axis of the plate. Finally, the material was heated with hot air to induce the reverse MT, studying the recovery strain due to the shape memory effect.

## 4. Results and Discussion

### 4.1. Microstructural Characterization

**Figure 4:**
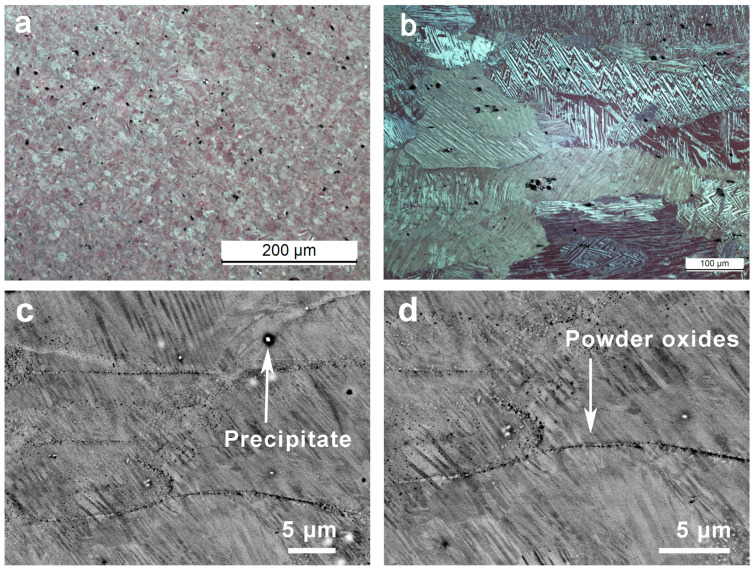
Microstructure of the Cu–Al–Ni HR sample. (**a**) Optical microscope image of the alloy after HIP but before the lamination process. (**b**) Optical microscope image depicting the elongation of the grains along the direction of the lamination (x direction). (**c**,**d**) SEM (BSE) images taken at 20 KV showing some precipitates at the grain boundaries as well as the oxide nanoparticles coming from the surface of the powder particles, which remain decorating the grain boundaries.

The obtained microstructure for the alloy is presented in Figure 4. Figure 4a depicts the state of the material after the HIP process, showing that the grains have a mean size smaller than 30 μm. From the optical image presented in Figure 4b for the material after the HR, it can be observed that the grains are no longer spherical and tend to elongate along the direction of the lamination process, exhibiting a noticeable growth, with sizes between 40 and 500 μm, that can be attributed to the dynamic recrystallization during hot rolling. On the other hand, even though the atomization of the powders takes place under an Ar atmosphere, the formation of a thin layer of oxide on the surface of the powder is inevitable. For this reason, when the particles are compacted during the HIP process, these oxides are trapped inside the material. Later, during the HR process, these layers of oxides are broken and distribute through the material as thin lines inside the material aligned along the rolling direction. Actually, this effect can be observed with backscattered electrons (BSEs) in SEM as in Figure 4c,d. However, these inclusions do not hinder completely the recrystallization processes or the evolution of the martensite plates during the martensitic transformation [120]. As an inconvenience, the sample also presents a certain amount of precipitate caused by the presence of the Zr grain refiner, which is depleted from the solid solution during the high-temperature processing by HIP and hot rolling and eventually coalesces during the further thermal treatments. Along the work, we realize that whereas the B seems to be a good grain refiner, in agreement with previous works [90,91], the Zr loses the refining performance during the processing at high temperatures between 850 and 900 °C, as will be shown through the EBSD patterns.

**Figure 5:**
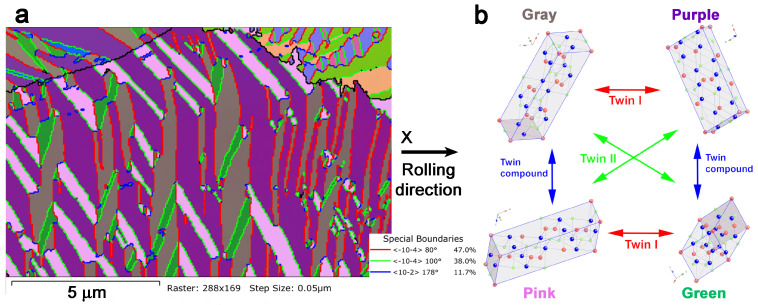
(**a**) EBSD map of the Cu–Al–Ni HR sample taken at room temperature in martensite, with a step size of 50 nm. (**b**) All variants correspond to the β’_3_ martensite, with the orientations indicated in the figures. The red interfaces are twin I, the greens are twin II, and the blues are the twin compound.

The EBSD measurements performed on the HR sample, see Figure 4b, show that the material is fully transformed into martensite at room temperature, and the EBSD maps show that all variants are indexed as the monoclinic β’_3_ martensitic phase (C2/m) (with a = 1.3817 nm, b = 0.52856 nm, c = 0.43987 nm, β = 113.6°) [127]. As an example, the EBSD map of Figure 5a presents the different martensite variants, and the black lines indicate two grain boundaries with more than 10 degrees of disorientation. The martensites form self-accommodated groups of four variants, whose orientations and orientation relationships (ORs) are determined by the EBSD indexations. The different colors of the martensite variants (gray, purple, pink, and green) correspond to the orientations given by the three Euler angles. The colors of the interfaces in Figure 5a correspond to the different twin boundaries, twin I and twin II, and the twin compounds. In Figure 5b, the orientation relationships of the unit cells are represented in the same colors of the map in the biggest grain of Figure 5a. These martensite variants accommodate according to three different twinning types that are indicated by arrows in the schema of Figure 5b in agreement with a previous description [128]; the special boundaries in between the variants and their percentages are also indicated in Figure 5a.

**Figure 6:**
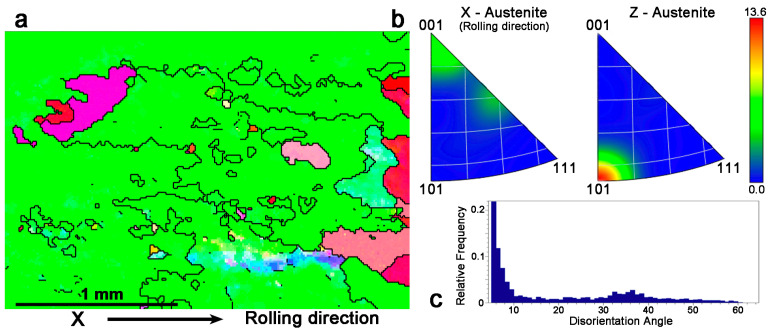
(**a**) EBSD map of the Cu–Al–Ni HR sample taken at 145 °C in austenite where the high-angle grain boundaries (θ ≥ 10°) are plotted; some boundaries are not closed because the disorientations below 10° are not plotted. (**b**) Inverse pole figures showing the crystallographic orientation densities (in arbitrary units on the right bar) corresponding to the rolling direction (X axis) and to the perpendicular to the rolling plane (Z axis). (**c**) Plot of the relative frequency of the grain boundaries as a function of their disorientation.

Once the material was characterized at room temperature and the martensites were identified, the sample was heated in situ inside the SEM using the heating stage to retransform the material to austenite and stabilize the temperature at 145 °C. This way, the austenite grains were studied by EBSD in many points of the HR plate. Figure 6a shows one of these images with the X axis on the rolling direction and the Z axis perpendicular to the paper. We can see very few grains, because in spite of the use of grain refiners, the hot rolling at 850 °C produced a dynamic recrystallization. In fact, we realized that at so high a temperature, the Zr agglomerates in small precipitates, losing or decreasing its performance as a grain refiner. Nevertheless, the grains become strongly textured with a preferential orientation of the [001] crystallographic direction in the rolling direction, as evidenced in the left side of the inverse pole figure in Figure 6b, and a preferential [101] direction on the Z axis, which is perpendicular to the rolling plane, as shown in the right inverse pole figure of Figure 6b. The analysis of the grain boundaries disorientation presented in Figure 6c shows that the grain boundaries drawn in black in Figure 6a have a main disorientation of about 30 to 40 degrees. However, the histogram shows that there is an extremely high density of low-angle disorientation sub-boundaries below 10 degrees, which are not plotted in Figure 6a for clarity because they really fill the image.

The same results were obtained in several fields of observation, and it can be concluded that there is a clear texture on Cu–Al–Ni SMAs processed through the proposed powder metallurgy route that was not previously reported. This texture could have an influence on the thermomechanical properties of these SMAs, because in Cu–Al–Ni single crystals, the higher transformation strain is measured for the [001] crystalline orientation [21,78].

### 4.2. Thermal Characterization

**Figure 7:**
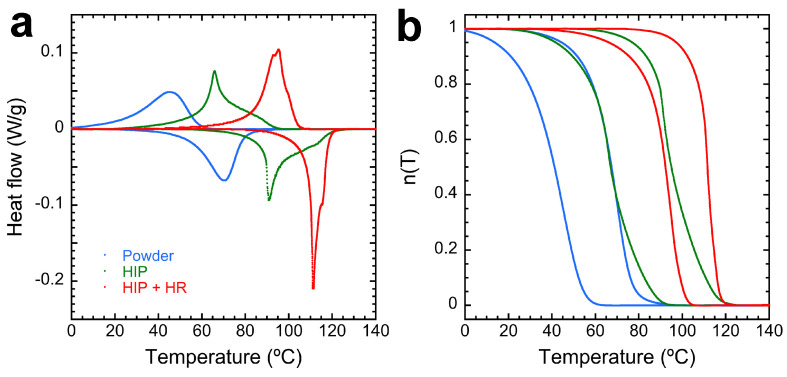
Transformation temperatures measured by DSC. (**a**) Transformation curves for the alloyed powders (in blue), HIPed samples (in green), and HR samples (in red). (**b**) Transformed martensite fraction for the curves presented in (**a**).

The thermal transformation of the Cu–Al–Ni sample is depicted in Figure 7. Figure 7a gathers the DSC curves of the atomized powders, the HIPed alloy, and the final HR sample. From these curves, the transformed martensite fraction (Figure 7b) is calculated to obtain the transformation temperatures, which are presented in Table 2. From these results, some important information can be derived. A noteworthy shift to higher temperatures is clearly observed in the processed samples compared with the original powders. It is a general observation performed in Cu-based SMAs produced by powder metallurgy [96,97,98], and the explanation is associated with the influence of the internal stresses on the martensitic transformation. The powder particles solidify in a very relaxed condition, even with a high cooling rate, because of the large free surface with respect to the volume, and consequently, the MT takes place in a low-stress condition. This means that the MT is dominated by the thermal transformation, taking place at lower temperatures. However, both the HIP and the HR processes induce high stress levels over the material, producing a shift in the transformation to higher temperatures. The influence of the internal stresses and their relaxation on the MT in Cu–Al–Ni alloys was quantified through adiabatic calorimetry and neutron diffraction [8,9].

### 4.3. Functional Behavior

**Figure 8:**
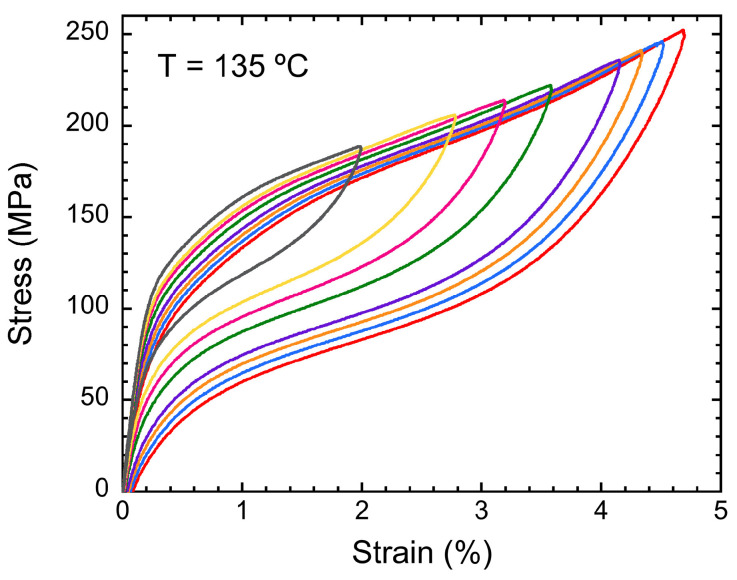
Tensile superelastic effect observed for the hot-rolled sample at different values of the maximum strain, up to 4.75%.

The thermomechanical properties of the SMA have been studied through tensile experiments of the SE. For the mechanical properties of the HR samples, a 4.75% recoverable strain was achieved in tension tests, as shown in Figure 8, with less than a 0.1% residual deformation. These measurements of the SE were conducted at 135 °C, a temperature value above Af, to ensure that the sample was completely in austenite. These results show an improved superelastic behavior in comparison with previous results in samples of the same family [117], exhibiting higher critical stresses as well as slightly higher maximum recoverable superelastic strain. This last result could be attributed to the preferential [001] texture obtained in the rolling direction, which is the tensile direction of the tested sample.

**Figure 9:**
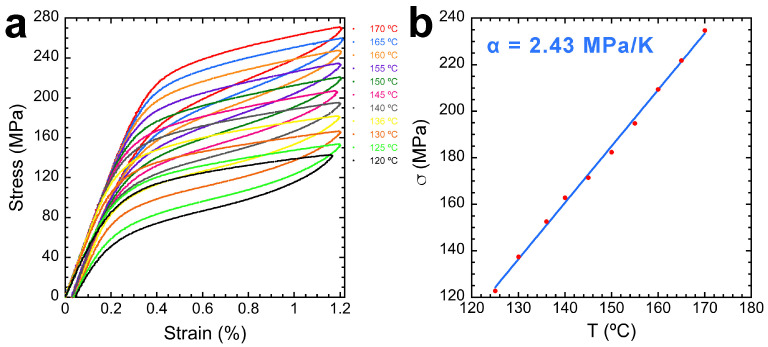
(**a**) SE at different temperatures, from 120 up to 170 °C every 5 °C, at 1.2% maximum strain. (**b**) Clausius–Clapeyron coefficient fitted from the data obtained in (**a**).

In Figure 9a, the evolution of the superelastic cycle is studied as a function of the experimental temperature. All cycles were carried out for a 1.2% maximum strain value. As the temperature increases, so does the critical stress σ_c_ for the stress-induced martensitic transformation. In single-crystal samples, this value can be clearly determined as the change in the slope is very steep. However, due to the high anisotropy of Cu–Al–Ni SMA [79,80,81], in the produced polycrystalline samples, each orientation presents a different value for σ_c_. For this reason, we observe a curve with an almost constant hardening as the transformation progresses.

In the present case, the numerical value for σ_c_ was determined by calculating the cut point between the straight line that arises by extrapolating the elastic stress–strain curve and the tangent straight line obtained from the plateau of the stress-induced MT. Furthermore, the evolution of σ_c_ on temperature is a very interesting property of thermoelastic SMA, commonly referred to as the Clausius–Clapeyron coefficient, α. This linear relationship can be fitted using the results of the experiments at different temperatures, giving a value of α = 2.43 MPa/K, represented in Figure 9b. This result of the Clausius–Clapeyron coefficient is very similar to, although slightly higher than, other results obtained for single crystals of alloys with similar compositions [21]. However, it is worthy of remark the fact that the hardening slope of the superelastic curve is not as high as could be expected for a polycrystal, and it could be also attributed to the [001] preferential texture in the rolling direction being also the one of the tensile tests.

**Figure 10:**
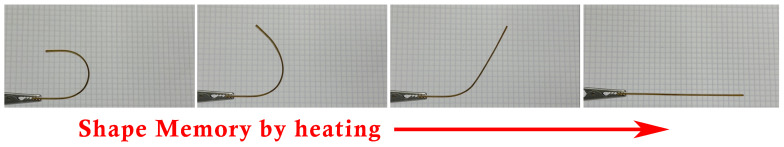
Sequence of the images showing the initial strained sample in martensite in the left image, and the evolution of the shape memory recovery by heating until the complete transformation to austenite, in the right image.

Additionally, a shape memory effect experiment was conducted using the laminated HR sample. The sample, indicated in Section 3.5, was deformed to a radius of 10 mm, corresponding up to ±5% strain with respect to the axis of the plate, as shown in the left image of Figure 10. Then, the material was heated with hot air to induce the reverse MT to austenite, recovering the 5% induced strain by the shape memory effect. The evolution of the reverse MT during the shape memory effect can be appreciated in the sequences of images in Figure 10. The experiment was repeated more than ten times with an exceptional reproducibility. The sequence of the shape memory recovery was recorded and is presented in Supplementary Video S1.

It is worthy of remark that the presented series of superelastic and shape memory results was obtained on polycrystalline materials of a Cu–Al–based SMA, which traditionally are considered as brittle alloys. This means that this powder metallurgy processing route allows overcoming such drawbacks and offers the possibility of taking the obtained performance of this PM SMA as reference material for the additive manufacturing processing methods producing Cu–Al-based polycrystalline SMAs. This will be particularly relevant because in the case of SMAs, their performance should be analyzed in terms of their functional properties.

## 5. Conclusions

In the present work, the complete processing route of a Cu-13.3Al-4.5Ni (wt%) shape memory alloy was approached from the powder production by gas atomization to the final processing through hot isostatic pressing and hot rolling. The alloy was designed to exhibit the martensitic transformation above room temperature. The martensitic transformation and the microstructure of the samples were studied alongside the production process, mentioning the heat treatments needed for the functionalization of the material. From the data extracted from the presented results, the conclusions can be summarized as follows:

- The presented powder metallurgy processing route based on pre-alloying before gas atomization, compaction by hot isostatic pressing (HIP), and hot rolling through small rolling steps, was revealed as an optimal process to improve the functional behavior of the alloy.
- Once the functionalization thermal treatments were applied to the material, the processed Cu–Al–Ni samples exhibited a highly reproducible martensitic transformation with a low hysteresis associated with the β’_3_ martensite.
- Even though polycrystalline Cu–Al–Ni SMAs tend to be fragile, the presented powder metallurgy processing route allowed obtaining samples with a specific [001] texture that contributed to their good functional properties.
- An impressive recoverable 4.75% superelastic deformation can be achieved in tensile experiments, as well as a ±5% fully recoverable shape memory effect, which was tested in bending.
- These outstanding results in polycrystalline Cu–Al–Ni SMAs pave the road for further advanced works and applications of these types of polycrystalline materials.

The present results validate the technique of powder metallurgy for Cu–Al–Ni SMAs and, in general, for any Cu–Al–based SMA, offering good functional performance that could be competitive with single crystals of similar alloys in applications where the maximum strain remains moderate. For this reason, we conclude that powder metallurgy can be an accessible technique for the design and fabrication of SMA components. The ability to modify and improve the microstructure and, therefore, the properties of the material with post-processing methods could be an advantage over the components processed by additive manufacturing. However, additive manufacturing techniques, like LPBF, EBM, or LMD, are producing polycrystalline near-net-shape components, and although such techniques have more limited post-processing capabilities, they could take the present powder metallurgy-produced material as a reference for the functional performances that could be obtained in polycrystalline SMAs produced by AM.

## Figures and Tables

**Table 1:** Obtained chemical composition measured by ICP and LECO for the powders obtained by gas atomization of the Cu–Al–Ni alloy.

Materials	Cu (wt.%)	Al (wt.%)	Ni (wt.%)	B (wt.%)	Zr (wt.%)	O ppm	N ppm	C ppm	S ppm
Target	82.2	13.3	4.5	0.2	0.1	-	-	-	-
Obtained	81.95	13.45	4.6	0.2	0.1	254	1	344	1

**Table 2:** Transformation temperatures and hysteresis from the n(T) curves shown in Figure 7.

CANBZ	°C				
	Ms	Mf	As	Af	ΔT
HIP + HR	102.3	55.0	90.9	118.4	19.3
HIP	89.5	33.5	69.1	117.5	28.0
Powder	57.2	6.6	39.0	82.6	26.4

## Data Availability

The original contributions presented in the study are included in the article/Supplementary Material, further inquiries can be directed to the corresponding author.

## Supplementary Materials

The following supporting information can be downloaded at: https://www.mdpi.com/article/10.3390/ma17246165/s1. Video S1: Shape memory recovery of the sample produced by powder metallurgy.
